# Comparative Structural and Computational Analysis Supports Eighteen Cellulose Synthases in the Plant Cellulose Synthesis Complex

**DOI:** 10.1038/srep28696

**Published:** 2016-06-27

**Authors:** B. Tracy Nixon, Katayoun Mansouri, Abhishek Singh, Juan Du, Jonathan K. Davis, Jung-Goo Lee, Erin Slabaugh, Venu Gopal Vandavasi, Hugh O’Neill, Eric M. Roberts, Alison W. Roberts, Yaroslava G. Yingling, Candace H. Haigler

**Affiliations:** 1 Department of Biochemistry and Molecular Biology, Pennsylvania State University, State College, PA 16802, USA; 2 Department of Crop Science and Department of Plant and Microbial Biology, North Carolina State University, Raleigh, NC 27695, USA; 3 Department of Materials Science and Engineering, North Carolina State University, Raleigh, NC 27695, USA; 4 Oak Ridge National Laboratory, Oak Ridge, TN 37831, USA; 5 Department of Biology, Rhode Island College, Providence, RI 02908, USA; 6 Department of Biological Sciences, University of Rhode Island, Kingston, RI 02881, USA

## Abstract

A six-lobed membrane spanning cellulose synthesis complex (CSC) containing multiple cellulose synthase (CESA) glycosyltransferases mediates cellulose microfibril formation. The number of CESAs in the CSC has been debated for decades in light of changing estimates of the diameter of the smallest microfibril formed from the β-1,4 glucan chains synthesized by one CSC. We obtained more direct evidence through generating improved transmission electron microscopy (TEM) images and image averages of the rosette-type CSC, revealing the frequent triangularity and average cross-sectional area in the plasma membrane of its individual lobes. Trimeric oligomers of two alternative CESA computational models corresponded well with individual lobe geometry. A six-fold assembly of the trimeric computational oligomer had the lowest potential energy per monomer and was consistent with rosette CSC morphology. Negative stain TEM and image averaging showed the triangularity of a recombinant CESA cytosolic domain, consistent with previous modeling of its trimeric nature from small angle scattering (SAXS) data. Six trimeric SAXS models nearly filled the space below an average FF-TEM image of the rosette CSC. In summary, the multifaceted data support a rosette CSC with 18 CESAs that mediates the synthesis of a fundamental microfibril composed of 18 glucan chains.

Cellulose within plant cell walls exists as semi-crystalline fibrils that form through the coalescence of numerous high molecular weight β-1,4-glucan chains. Cellulose fibrils play critical roles in plant development by constraining the direction of cell expansion and conferring strength to the plant body. Cellulose also plays important roles in industrial products such as wood and paper. Plant cell walls, inclusive of cellulose and other polymers, serve as abundant renewable carbon storage reservoirs. Cell wall degradation is also important within natural ecosystem cycles, in animal feed, and to release sugars from lignocellulosic biomass during the production of biofuels^1^. A better understanding of the mechanisms of cellulose fibril formation would allow us to engineer cellulosic plant products for specific uses.

Cellulose microfibrils in the land plants and their close algal relatives are synthesized by a six-lobed ‘rosette’ cellulose synthesis complex, or rosette CSC. These distinctive multimeric transmembrane protein complexes are revealed by freeze fracture electron microscopy (FF-TEM)^2^,^3^. In FF-TEM, frozen specimens are cleaved, which often splits the two membrane leaflets. The specimen is then shadowed with a platinum/carbon (Pt/C) mixture so that the intramembrane proteins, including cellulose synthases (CESAs) within CSCs, become visible as particles on otherwise smooth membrane surfaces^4^. The transmembrane helices (TMHs) of numerous assembled CESAs form the ‘rosette’ shape in the FF-TEM replica (Fig. 1), as confirmed by immunolabeling^5^. The CESAs are glycosyltransferases that use UDP-glucose substrate to synthesize a single glucan chain, as shown by structural comparisons between CESA and BcsA, a bacterial cellulose synthase^6^,^7^. The assembly of multiple CESAs into one CSC results in many glucan chains being synthesized in close proximity to facilitate microfibril formation near the extracellular surface of the plasma membrane^8^.

A persistent question for decades has been: How many CESAs are in one rosette CSC, and, consequently, how many glucan chains form the fundamental cellulose fibril in plant cell walls? Although it has long been conjectured that 36 CESAs exist in one rosette CSC^9^, this idea has been questioned on several grounds. When the lobe area was compared to a typical cross-sectional area for one TMH in the context of 8 predicted TMHs in one CESA, a maximum of four CESAs per lobe (24 total CESAs) were proposed for the rosette CSC^10^. However, this analysis was limited by the use of a generic estimate of TMH area and images of rosette CSCs after shadowing with a thick coating applied unidirectionally from a 45° angle. This traditional FF-TEM method resulted in the perception and measurement of the lobes in part through their electron transparent ‘shadows’ where metal was not present, leading to imprecise estimates of lobe shape and dimensions. Early electron diffraction data from cotton and rose primary walls were consistent with 12 to 25-chain cellulose fibrils^11^,^12^, and recently 18- or 24-chain fundamental fibrils have been favored based on spectroscopic analyses of three distinct cell wall types, as well as computational simulations alone or in reference to X-ray diffraction data^13^,^14^,^15^,^16^,^17^. Most recently, an 18-chain fundamental cellulose microfibril was inferred from the *in vitro* formation of a trimer from the catalytic domain of CESA1 from *Arabidopsis thaliana* (AtCESA1; GenBank NP_194967.1), which could represent the cytosolic component of one lobe of the rosette CSC^18^. The TMH were not present in the expressed AtCESA1 fragment, which presented the opportunity to use other methods to analyze the size and shape of the TMH region of the rosette CSC for direct comparison FF-TEM images. The recent biochemical evidence for 1:1:1 stoichiometry for the three CESA isoforms within both primary and secondary wall rosette CSCs^19^,^20^ is equally consistent with 18 or 36 CESAs (three or six per lobe), implying the need for additional types of data to test whether the rosette CSC might contain fewer than 36 CESAs.

Here we provide several types of evidence to define 18 CESAs as the maximum within one rosette CSC. We used improved FF-TEM methods^21^ and image averaging to establish an average size and typical shape for each lobe of the rosette CSC. Superior images were collected from *Physcomitrella patens*, a moss with dense rosette CSCs in growing protonema^22^, and compared with: (a) newly developed computational models for parts of *Gossypium hirsutum* (cotton) CESA (GhCESA1; GenBank AAB37766.1); and (b) previously published^18^ and new negative stain TEM structural information for the trimeric catalytic domain of AtCESA1. Even in diverse plant CESAs, both the predicted TMH regions and the large catalytic domain are highly similar (Supplementary Figs S1 and S2), which allowed the synergistic interpretation of data from different CESA isoforms to make size comparisons. In addition, we determined the dimensions and potential energies per monomer of various modeled oligomers of GhCESA1 (dimers through hexamers) and their six-fold assemblies for comparison to the rosette CSC morphology. The data refute the original model of 36 CESAs and support 18 CESAs within the rosette CSC, which is consequently predicted to synthesize an 18-chain fundamental cellulose fibril within cell walls of land plants and their closely related algal progenitors.

## Results

### Measurements of Rosette CSCs in Refined Original Images

**Figure 1:**
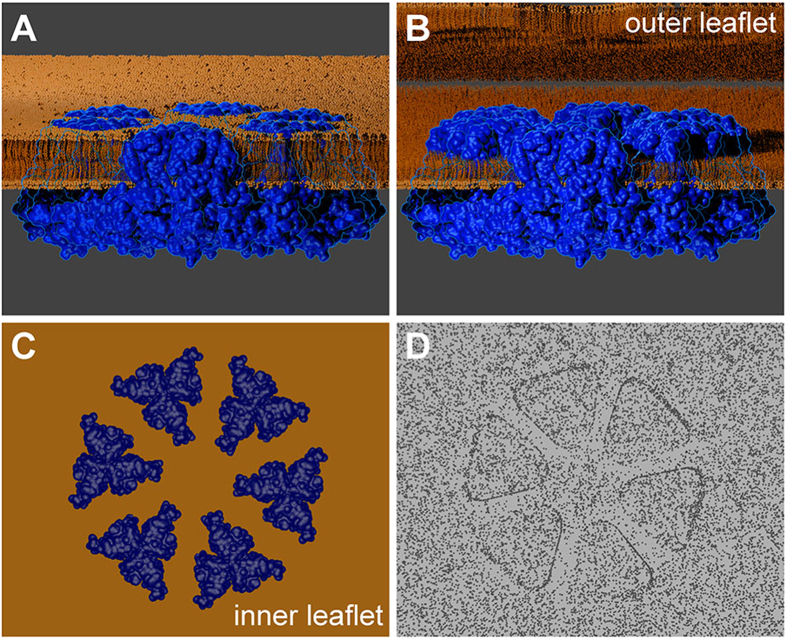
Cartoon to show how the TMH region of the CSC is viewed within replicas prepared by FF-TEM. The cartoon was based on the 7 TMH CESA model as described in Supplementary Methods. **(A)** The membrane-spanning CESAs (blue) within a rosette CSC are embedded in the intact plasma membrane bilayer (orange). The membrane is cut away to reveal one of six lobes in face view. The top of the TMH region emerges minimally on the surface of the plasma membrane^8^, and the catalytic domain is in the cytoplasm. **(B)** During specimen fracture, the outer leaflet of the plasma membrane is typically removed so that half of each ‘column’ of assembled TMH is revealed above the interior face of the inner plasma membrane leaflet (called the protoplasmic fracture, PF, face in FF-TEM terminology^58^). **(C)** A top-down view of the TMH of the rosette CSC, embedded within the inner leaflet of the plasma membrane, prior to metal shadowing and replication. The cytosolic portions of the assembled CESA proteins remain unseen beneath the membrane. **(D)** A representation of the metal replica that is finally viewed in the TEM after the removal of the biological material.

**Figure 2:**
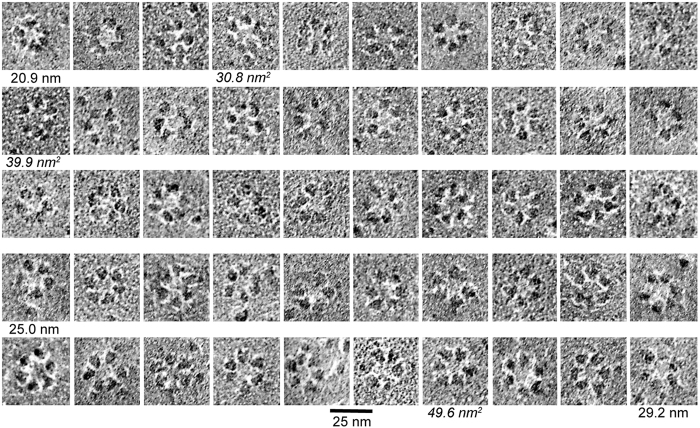
Gallery of fifty original FF-TEM images of rosette CSCs. The gallery is arranged in order of estimated circular diameter, from smallest (20.9 nm) to largest (29.2 nm) as labeled below the panels. A rosette CSC with circular diameter near the mean value (25.0 nm) for the original images is also labeled. Three rosette CSCs labeled with nm^2^ in italic text are those with the minimum, mean, and maximum values for individual lobe area, as measured in the original images and averaged across the six lobes of each CSC. The 25 nm scale bar applies to all images.

The rosette CSCs analyzed here reflect the top-down view of the assembled TMHs of multiple CESAs as revealed by FF-TEM (Fig. 1). Figure 2 shows a representative gallery of rosette CSCs in association with membrane surfaces without visible fibrillar elements, consistent with imaging of the plasma membrane inner leaflet where rosette CSCs are most often revealed in FF-TEM. The images have relatively low contrast due to the use of rotary shadowing from a 60^o^ angle. The white zones around some lobes are likely attributable to a ‘decoration effect’, or the preferential migration of Pt/C to structures that promote nucleation and growth of grains. This phenomenon leads to localized reduction of electron density in adjacent areas^4^.

Two methods of measuring perimeter of the replicated rosette CSCs on the original images yielded mean values of 74.8 nm (using hexagonal geometry) to 78.4 nm (using circular geometry) (Table 1). The circular estimate was 3.6 nm (4.8%) greater than the hexagonal estimate. The calculated mean diameter was 21.4 nm (using hexagonal geometry, with a 17.6–25.6 nm range) or 24.9 nm (using circular geometry, with a 20.8–29.2 nm range). Control analyses based on FF-TEM images and the solved structure of membrane-spanning aquaporin-4 showed that these measurements were not inflated by the metal shadow applied using our improved FF-TEM methods (Supplementary Fig. S3).

### Measurements of Entire Rosette CSCs and Individual Lobes in Class Average Images

**Figure 3:**
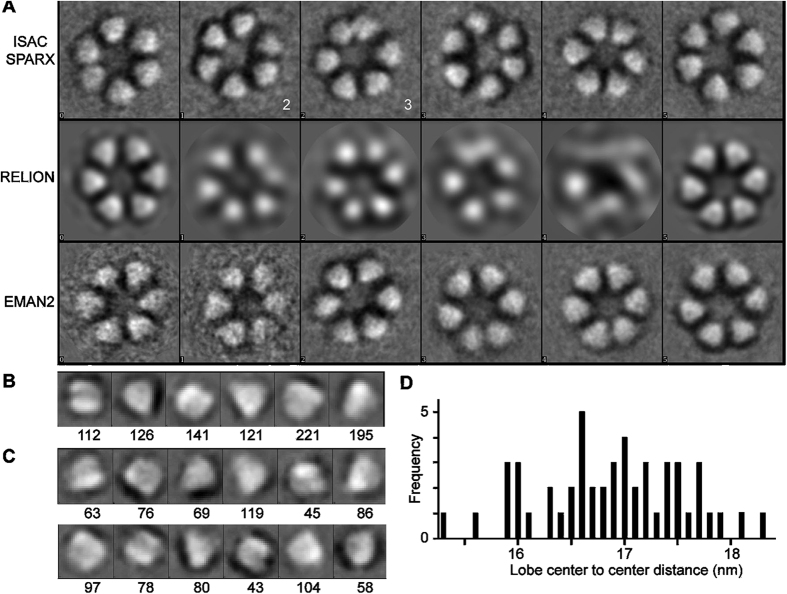
Class averages of rosette CSCs and individual lobes imaged by FF-TEM. **(A)** Class averages of entire rosette CSCs as derived from the three programs named on the left of each row. The white lobes arose from reversal of the original image contrast before averaging. ISAC/SPARX class average 2 was used for further comparisons because the spacing of its opposite lobes matched the average shown in **(D)**. ISAC/SPARX class average 3 shows two lobes close together at the top of the image. ISAC/SPARX gave 6 class averages, so the programs RELION and EMAN2 were also set to provide 6 classes. Each side of the box containing a rosette CSC = 31.6 nm. **(B)** Six or **(C)** 12 class averages as derived by EMAN2 from individual lobes are shown, with the number of lobes in each class indicated below each panel. Each side of the box containing a lobe = 10.6 nm. **(D)** The distribution of center-to-center distances between opposite lobes in the class averages of the rosette CSCs in (**A**).

Class averages of entire rosette CSCs and individual lobes were generated computationally (Fig. 3). From an initial set of 497 images of rosette CSCs with inverted image contrast, similar class averages were derived by use of three image averaging programs, ISAC/SPARX^23^, RELION^24^, and EMAN2^25^ (Fig. 3A). In the 14 clear class averages, the mean diameter of the rosette CSC was 23.6 nm, which was only 0.5 nm greater than the average from the original images (Table 1). In class averages of rosette CSCs with sharp contours, the lobes were most often separated with a distinct boundary (Fig. 3A). The placement of lobes around the perimeter was largely symmetric, although adjacent lobes were sometimes closer together (Fig. 3A, ISAC/SPARX class average 3). The radial distance between the centers of opposite lobes varied from 15.1 to 18.6 nm (averaging 17.0 ± 0.7 nm) (Fig. 3D). This is consistent with the elliptical appearance of some rosette CSCs. ISAC/SPARX class average 2 was used for further comparisons because its opposite lobes were spaced at the mean value (17.0 ± 0.36 nm).

The improved FF-TEM methods used here allowed a better assessment of lobe shape. In the original images (Fig. 2), some individual lobes appeared triangular with one vertex pointing to the center. Triangular lobes also appeared in class averages of entire rosette CSCs (Fig. 3A) and when individual lobes were picked and averaged separately (Fig. 3B,C). Based on the number of lobes in each class after averaging of individual lobes (shown below each panel in Fig. 3B,C), 30 to 70% of the lobes are ‘triangular’ depending on how strictly one visually defines a triangle. Intuitively, triangular shape is consistent with a trimeric lobe, which was evaluated further through comparison of imaged and modeled area estimates.

The average cross-sectional area of replicated individual rosette lobes, as measured by tracing them in the original images, was 39.9 ± 6.5 nm^2^ (Table 1; Fig. 2), with a range of 24.8–62.1 nm^2^ within the 300 individual measurements. A similar average area (39.1 nm^2^) was derived from class averages of entire rosette CSCs or six or 12 classes of 916 individual lobes picked separately (Table 1). These area estimates correspond to an equilateral triangle with about 8 nm height—near the diameter previously ascribed to each lobe of the rosette CSC based on low precision visualization in FF-TEM replicas prepared by unidirectional shadowing from a 45° angle^26^,^27^.

### Comparison of Rosette CSC Images with Modeled CESA TMH Regions

In order to assess the plausible number of CESA monomers in the lobes of rosette CSCs, we compared the cross-sectional area occupied by different oligomers of modeled TMH regions with the lobe area estimates derived from FF-TEM images of rosette CSCs.

#### Comparison of an 8 TMH model

**Figure 4:**
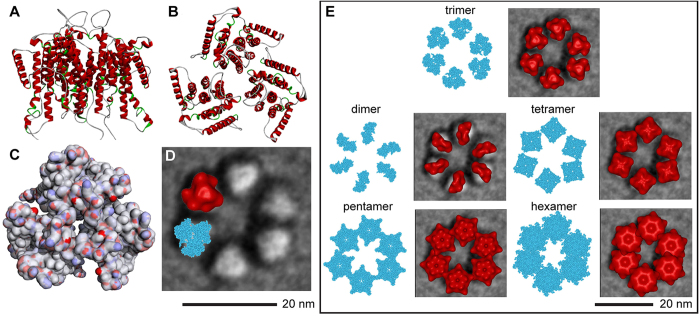
Computational prediction of a trimer of the putative 8 TMH region and spatial comparison to the rosette CSC. In this analysis, only 8 TMH were included and the oligomers were centered manually on the lobes of the rosette CSC. **(A)** View from the side of 24 total alpha helices (red), eight within the TMH region of each CESA monomer. **(B)** View from the top (outside the cell). **(C)** Solvent accessible surface, as viewed from the top, colored by electrostatic potential. Blue, white or red represent positive, neutral charge, or negative charge, respectively. **(D)** A representative class average image of the rosette CSC (ISAC/SPARX class average 2 from Fig. 3) overlaid with the computed trimer of the TMH region depicted as Van der Waal spheres (cyan) or as an isosurface of the structure blurred to 2.5 nm resolution (red). A 20 nm scale bar for this panel is shown below. **(E)** The various oligomers were replicated six times (shown separately in cyan representations of atomic van der Waals spheres) and overlaid on the ISAC/SPARX class average 2 image of the rosette CSC. In the overlays, the atomic models are rendered as isosurfaces and shown in red. All views are ‘top down’, as if viewed from outside the cell. A 20 nm scale bar for this panel is in the lower right.

Having observed triangular lobes in single particle and class average images, we generated an *in silico* trimer from the modeled 8 TMH region of a plant CESA (Fig. 4). The monomeric ‘8 TMH’ model reflected long-standing predictions about CESA membrane topology^4^. The 323 amino acids that were modeled (Supplementary Fig. S4) represented only the putative TMH of GhCESA1 linked together by short amino acid regions, including an artificial one to replace the large central domain. As explained further in Supplementary Methods, the RaptorX server ( http://raptorx.uchicago.edu) was used to select templates that were best matches to different parts of the CESA TMH region and perform homology modeling (Supplementary Table S1). Due to limited sequence homology in the TMH region between BcsA and CESA, RaptorX did not identify BcsA as a template during production of the 8 TMH model (Supplementary Fig. S5). The selected 8 TMH monomeric model was refined by molecular dynamics (MD) in the presence of a simulated lipid bilayer (Supplementary Fig. S6) prior to docking to form various oligomers.

The modeled 8 TMH trimer was reasonably tightly packed (Fig. 4A–C), with a total cross-sectional area of 41.9 nm^2^ that was only 6% larger than the average lobe area derived from imaging (Table 1). The trimeric oligomer was the best fit with individual lobes of ISAC/SPARX class average 2 (Fig. 4D). To the contrary, the dimer did not fill a lobe and the tetramer extended beyond the lobe edge into the space between them. The pentamer and the hexamer clashed with their neighbors when manually centered on the six lobes of ISAC/SPARX class average 2, which had the average spacing between opposite lobes (Fig. 4E). The total cross-sectional areas in the other oligomers of the 8 TMH model were determined to quantitate these visual impressions. As compared to the 39.5 nm^2^ overall mean lobe area derived from images (Table 1), the dimer model occupied too little area, and the tetramer, pentamer, and hexamer models occupied too much area (Table 2).

#### Comparison of a 7 TMH CESA model

Putative TMH 5 of CESA could instead lie on the inside of the plasma membrane so that a maximum of seven TMHs exist in CESA, placing a substrate binding loop in the cytoplasm of CESA as occurs in bacterial BcsA^28^,^29^. We considered this possibility to be likely given that binding of substrate is critical for catalysis and important related residues in BcsA (within an FxVTxK motif) are conserved across Kingdoms^28^. Therefore, an alternative model with seven TMHs and structural similarity to BcsA was generated by manually specifying BcsA as a template. This 7 TMH region was docked to a predicted structure of the GhCESA1 cytosolic domain, which had optimized folding of two plant-specific domains in CESA, the plant conserved region (P-CR) and class specific region (CSR)^7^. The P-CR and the CSR are on the periphery of the catalytic core, which retained the same structure in the optimized model^7^ as in the first version of the CESA model^30^. This structural prediction is referred to hereafter as the ‘7 TMH CESA’ model, and it has the putative substrate-binding loop on the cytosolic side.

The monomeric 7 TMH CESA model in lipid was subjected to MD refinement by the same methods described for the 8 TMH model. Then dimeric through hexameric oligomers were generated, computationally refined, and analyzed in terms of TMH cross-sectional area and potential energy (Supplementary Table S2; Table 2; Table 3). Analysis of the potential energies was anticipated to be more meaningful for the 7 TMH CESA model, due to the inclusion of the large central catalytic domain. The overall shape of each oligomer was similar to those shown for the 8 TMH model (compare the images in Supplementary Table S2 to those in Fig. 4E). The cross-sectional area of the modeled seven TMH region (44.4 nm^2^, Table 2) was again the closest match to the 39.5 nm^2^ overall mean lobe area derived from images (Table 1). Within the set of potential energies per monomer, the trimer had the second lowest energy (−1.65 kcal/mol × 10^−5^), which was close to the lowest value for the hexamer (−1.69 kcal/mol × 10^−5^).

**Figure 5:**
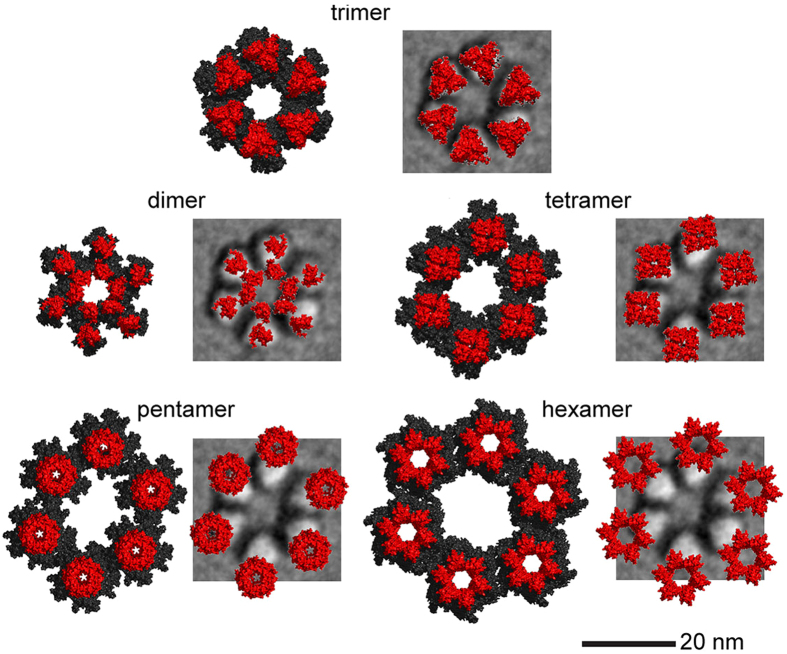
Computational predictions of the various oligomers of the putative 7 TMH CESA model and spatial comparisons to the rosette CSC. In this analysis, seven TMH and the large catalytic/cytosolic domain were included in the model, and the oligomers were refined in MD simulations. All views are ‘top down’, from outside the cell. For each modeled oligomer (see labels in the figure), the left member of the pair shows the catalytic/cytosolic domain in black and the TMH in red. The right member of the pair shows only the TMH region overlaid on the ISAC/SPARX class average 2 image of the rosette CSC. The six-fold assembly of the trimeric TMH has the best fit with both the diameter and triangular lobe shape of the FF-TEM average image. The 20 nm scale bar applies to all images.

Six-fold assemblies of the various 7 TMH CESA modeled oligomers were generated to mimic the organization of the entire rosette CSC and refined in MD simulations. Among these, the assembly of trimers had the lowest energy per monomer (−2.50 kcal/mol × 10^−5^) (Table 3), and the placement of the TMH regions had the best correspondence to the average FF-TEM image (see red areas in the trimer in Fig. 5). The cytosolic side (represented in black) of the computational six-fold assembly of predicted trimeric oligomers formed a continuous ring. The average diameter of the cytosolic portion was 32.8 nm, as averaged from three pairwise measurements between the outer edges of opposite lobes.

### Demonstration of Triangularity in a CESA Cystosolic Domain Trimer and Comparison of its SAXS-derived Model to the Rosette CSC

**Figure 6:**
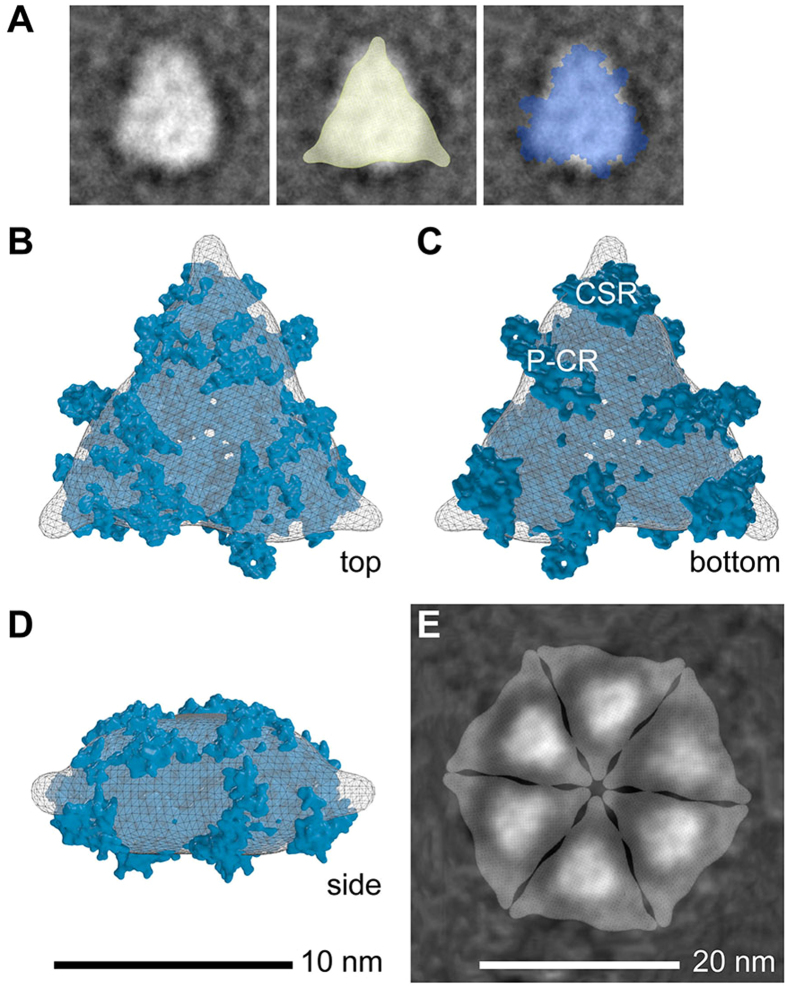
Comparison of images and models shows that a maximum of 18 CESAs can exist within the rosette CSC. **(A)** A class average image of the negative stained trimeric AtCESA1 cystolic domain (left panel) with a cross-sectional area close to the mean value (108.7 nm^2^; see Supplementary Fig. S7). The class average image was superimposed with the SAXS filtered average model (middle panel; yellow triangle; 100 nm^2^ cross-sectional area) or a flat rendering of the cytosolic portion of the trimeric 7 TMH CESA model (right panel, blue molecular shape; 104 nm^2^ cross-sectional area). **(B–D)** Top, bottom and side views of the SAXS volume in (A) superimposed with the cytosolic domain of the trimeric 7 TMH CESA model. The 10 nm scale bar applies to panels B-D. **(E)** Six-fold replication of a semi-transparent rendering of the trimeric SAXS model overlaid onto an average FF-TEM image of the assembled TMH regions of the rosette CSC (ISAC/SPARK class average 2). This schematic represents the rosette CSC structure as if viewed from the cytosolic side. A 20 nm scale bar for this panel is shown.

Previously a trimeric model of the purified cytosolic domain of AtCESA1 was generated from SAXS data with GASBOR^18^. To validate the model, we used negative stain TEM to visualize the recombinant protein solution, picked 138 trimer-sized particles many of which appeared triangular, and used EMAN2 to generate six class average images that also frequently appeared triangular (Supplementary Fig. S7). For further comparisons, we used the negative stain image average with area near the mean of the set of six (108.7 ± 10.8 nm^2^), as shown in Fig. 6. This particle (Fig. 6A) was only 4% to 8% larger than the maximum cross-sectional areas of the CESA cytosolic trimeric models generated by purely computational methods (104.1 nm^2^) or from the SAXS data (99.6 nm^2^; see the overlays in Fig. 6A).

When the average filtered SAXS model was superimposed with the cytosolic portion of the 7 TMH CESA model by use of SUPCOMB^31^, similar shapes were observed (Fig. 6B–D). The structural superimposition had a normalized spatial discrepancy (NSD) of 0.95, indicative of a reasonable fit. (An NSD of 0 indicates ideal superimposition and values above 1 indicate systematic structural differences^31^.)

The SAXS model was replicated six times and compared to the average FF-TEM image by symmetric manual arrangement with close packing and minimal overlap. The SAXS model nearly filled the space marked out by the TMH domains of the rosette CSC, and each TMH lobe was approximately in the center of the SAXS model (Fig. 6E). This composite image and the hexamer of trimers of the 7 TMH CESA model (Fig. 5) have differences in details and are not expected to represent the spatial relationships in the rosette CSC precisely. These schematics do illustrate a high degree of compatibility between the shapes and dimensions of modeled trimeric CESA TMH and cytosolic regions, individual lobes, and an entire rosette CSC assembled from six trimeric lobes.

## Discussion

These multifaceted data strongly support a maximum of 18 CESAs in the rosette CSC. We compared its accurately-estimated size and shape with two newly developed computational models and a previously published triangular trimeric model of the CESA cytosolic domain based on SAXS data^18^, which we validated here through negative staining. Triangular lobes of rosette CSC were observed frequently in original and averaged FF-TEM images. This observation relied on more precise images produced with optimized FF-TEM methods^21^ that: minimized protein distortion upon fracture; nearly eliminated molecular surface contamination after fracture; covered the specimen with a minimal amount of shadowing metal; and reduced the Pt/C grain size so that the replicas portrayed greater biological detail. In addition, when compared to conventional 45° unidirectional Pt-C shadowing, rotary 60° shadowing coated the specimen more evenly and facilitated more accurate measurements^32^. The vertex pointing to the center in the average images of entire rosette CSCs is consistent with recent speculative diagrams showing how one CESA isomer, of the three isomers believed to exist within each lobe, can be consistently oriented toward the center of the rosette CSC in an arrangement that limits the multi-protein complex to six lobes^15^,^20^. It also remains possible that the individual lobes of the rosette CSC are homomeric, with three different CESA isomers at least sometimes existing within different lobes of the same CSC^18^ or, an entirely homomeric CSC may also exist in nature.

The GhCESA1 models used here are part of our ongoing efforts to generate a complete CESA model, as well as to explore the implications *in silico* of seven or eight TMH existing in CESA. The 7 TMH CESA model and the recombinant AtCESA1 cytosolic fragment both lacked the relatively short C-terminal region (about 20 amino acids) and the N-terminal region (typically 150–250 amino acids), which is likely to exist in the cytoplasm. Additional structural information is required before the flexible and variable N-terminal region can be included in the *ab initio* CESA model, and an experimentally-determined topology of CESA has not been yet been published. Nonetheless, the partial models were sufficient for making broad spatial comparisons to the rosette CSC images showing the TMH regions. Although the optimized FF-TEM images allowed excellent estimates of the size and shape of rosette CSCs to be made in the native membrane context, the images reflect the protein complex in the frozen state (0 K), whereas MD was performed at 300 K as is typical for biological molecules. By a small factor, this would tend to make the models larger than the particles. In addition, the cross-sectional areas of the models may differ slightly from reality due to factors such as: (a) use of dioleoyl-phosphocholine (DOPC) lipid instead of the unknown native lipids surrounding the rosette CSC; (b) not including the large catalytic domain in the 8 TMH model; and (c) the assembly of oligomers from one CESA isoform instead of three isoforms as may occur *in vivo*^19^,^20^. Despite these caveats that could have small effects on the measured cross-sectional areas, the synthesis of all the data is consistent with each single lobe of the rosette CSC containing three CESAs.

Among the diverse oligomers tested, the cross-sectional areas of the trimeric isolated 8 TMH model and the TMH portion of the more complete 7 TMH CESA model were closest to the average lobe area derived from imaging. The predicted trimeric cytosolic domain structure was a good fit with a class average image of the negatively stained recombinant CESA cytosolic domain trimer, as previously modeled based on SAXS data^18^, and it had a low potential energy. The six-fold assembly of the trimeric oligomer of the 7 TMH CESA model had markedly lower energy as compared to the other oligomers. After refinement by MD in the presence of explicit water, this six-fold assembly had the best fit with the size and shape of the average FF-TEM rosette CSC image. These data contrast with the mixture of monomeric and dimeric forms reported for the heterologously expressed cytosolic domain of a rice CESA^33^. Although CESAs have similar size so that spatial comparisons of equivalent oligomers can be made between different isoforms, the isoforms may have differences in interaction potential that are potentially also affected by experimental conditions as discussed previously^18^.

Both the SymmDock and SAXS trimeric models lacked the N- and C-terminal CESA regions and lipid in the system. Detailed interpretation of the placement of CESA domains will be most appropriate when complete models are available and/or when experimental evidence is available to test the predictions. Preliminarily, for the 7 TMH CESA model we note that the substrate-binding region is on the cytosolic side, and this may in fact be the native conformation of CESA. In this case, the substrate could be stabilized in the catalytic site of CESA similarly to BcsA, but this needs to be further tested through determination of CESA membrane topology and other experiments. Regarding the placement of the plant-specific regions of CESA, the CSR region lies within the three vertices of the triangular homomeric lobe and the two parallel helices of the P-CR region extend inward on cytosolic side of the predicted trimer (remote from the TMH region). A similar arrangement for the plant-specific domains of CESA was previously described for the SAXS cytosolic model^18^. The SymmDock six-fold assembly of trimers had an estimated diameter on the cytosolic side of 32.8 nm, which is 21% larger than the 27 nm maximum diameter of the six-fold manually produced schematic of the SAXS model^18^ as measured where the vertices of the trimeric cytosolic domains of adjacent lobes approach each other (Fig. 6E). Determining whether the central space on the cytosolic side exists or not requires empirical information about the structure of the entire CSC. The average 30 nm predicted cytosolic diameter of the rosette CSC is 28% larger than the 23.4 nm average diameter of the TMH region as seen in FF-TEM images (Table 1). A putative 30 nm cytosolic diameter for the rosette CSC is considerably smaller than the 45–50 nm diameter structures previously proposed to represent this side of the complex^10^. However, the current data do not take into account the spatial contributions of the N-terminal domain and accessory proteins that are likely to associate with the rosette CSC on the cytosolic side.

Despite the strong support provided by our data for a trimeric lobe in the rosette CSC, we considered the range of lobe areas: 33 to 46 nm^2^ in the class averages of individual lobes and 25 to 62 nm^2^ in original images. This range of values could derive from biological variation (non-equivalency in different lobes). For example, two proteins each predicted to contain a single TMH are associated with the active CSC in the plasma membrane, KORRIGAN1 and COMPANION OF CELLULOSE SYNTHASE^34^,^35^,^36^. Although the stoichiometry of any accessory protein in the CSC is unknown, they could increase the lobe cross-sectional area and/or cause deviations away from triangular morphology in some or all lobes. Alternatively, the image contrast and detail of FF-TEM images is generated through the accumulation of metal grains (>1.25 nm diameter in these replicas) on the biological structures. Grains of metal could form on the side of the actual protein structure and expand the natural shape artificially, although this effect is not pronounced as determined through analysis of the AQP4 control. Confirmation of any natural variation between lobes awaits high resolution structural analysis of isolated rosette CSCs.

Rosette CSCs had variable diameters, which suggests that connections between the individual lobes are relatively weak. The presence of weak lobe-lobe interactions was also indicated for the homomeric trimers of the AtCESA1 catalytic domain - no signs of higher order oligomers were evident in dynamic light scattering and solution X-ray scattering experiments^18^. In the FF-TEM images, two lobes sometimes approach each other more closely than typical adjacent pairs. We infer that the forces that hold the rosette CSC together must occur below or above the TMH regions as mediated by protein/protein interactions inside the cell and/or glucan chain interactions near the plasma membrane surface. For example, changes in conformation of the cytosolic portion of CESA or the association of a regulatory protein with the complex could affect the spacing between adjacent lobes. Computational modeling supports the idea that the polymerization and crystallization of cellulose can transmit forces to the rosette CSC complex sufficient to account for its observed movement in the plane of the plasma membrane^37^. Possibly, variable activity states of the rosette CSC are associated with changes in the complex diameter and/or other aspects of its detailed morphology as observed here and in prior FF-TEM images^27^.

For an 18-chain cellulose microfibril, only 6 chains are predicted to be in the interior away from immediate interaction with solvent^15^,^16^,^17^. An 18 chain fibril may represent the minimum diameter that can maintain an extended linear form within a hydrated apoplastic space inclusive of cellulose-interacting matrix components. Importantly, 18-chain cellulose fibrils may not finally exist, or exclusively exist, within plant cell walls. Consistent with experimental evidence^14^,^15^,^16^, larger crystalline cellulose fibrils may often form as adjacent 18-chain fibrils coalesce and co-crystallize to form larger cellulose macrofibrils. The characteristics of 18-chain cellulose fibril may promote adaptive variation in final cell wall architecture. Fibrils in this size range have good longitudinal but poor lateral chain order^11^,^12^,^38^, and loosely organized chains may facilitate cellulose-cellulose or cellulose-matrix interactions within diverse cell wall networks.

Numerous algae have rectangular CSCs associated with the synthesis of large microfibrils, e.g. the cellulose fibrils in *Valonia ventricosa* contain about 1200 glucan chains^39^. The marked change to the rosette CSC morphology that occurred along with the transition of plants to land, with the concomitant reduction in the size of the fundamental cellulose fibril to one containing 18 chains, may have been an adaptive feature conferring more possibilities for the final size of cellulose fibrils and cell wall structure in parallel with new biophysical constraints and increasing anatomical diversity as plants adapted to terrestrial environments. The future engineering of CSC composition and structure could, in turn, lead to cellulose fibrils with different size, shape, and crystalline properties within cellulosic biomaterials tailored for particular uses.

## Methods

### Culturing Moss and Imaging Rosette CSCs

#### Culturing moss and preparing samples for FF-TEM

*P. patens* was sub-cultured and grown five days in a lighted 23 °C incubator on cellophane disks overlaying solid basal medium with ammonium tartrate^40^. An actively growing protonemal colony was mounted in 1 μl rehydrated yeast paste between two copper planchets, frozen within a few seconds in liquid-nitrogen-cooled resolidifying propane using an automated plunger (EMS 002 Rapid Immersion Freezer; Electron Microscopy Sciences, Hatfield PA), and stored in liquid nitrogen.

#### FF-TEM

Samples were loaded under liquid nitrogen into a ‘double replica’ specimen holder, which held the two planchets together until it was opened to fracture the specimens. The closed specimen holder was transferred onto the ultra-cold stage (−185 °C) under vacuum of a freeze fracture machine (Model 308R; Cressington Scientific Instruments, Watford, UK). The specimen stage had an ultra-cold shield to scavenge molecular contaminants^8^. Under low vacuum (<1 × 10^−7^ mbar), the stage was warmed to −120 °C for 10–15 min to evaporate excess propane then cooled to −160 °C. The electron beam evaporators were degassed (2–4 cycles; 1.5 kV/50 mA), then the specimens were fractured followed by immediate stage rotation (118 rpm) and Pt/C shadowing (1.4–1.5 nm; 60°). This film thickness was the minimum that resulted in a relatively continuous coating of the specimen. The replica was stabilized with carbon (13.0–15.0 nm; 85° angle). Replicas were floated onto 1 ml sterile water containing 2 drops of 1% Photo-Flo (Eastman Kodak Company, Rochester NY), cleaned for 2–3 h by floatation on chromic acid (7.8% w/v potassium dichromate in 33% v/v sulfuric acid), and rinsed in water plus Photo-Flo. Dried replicas on 75 mesh, Formvar-coated, copper grids were imaged in the TEM (JEOL-1200EXI, Tokyo, Japan). The microscope magnification was calibrated daily using crystals of bovine liver catalase (Ladd Research Industries, Williston VT) that had been negatively stained with 2% (w/v) aqueous uranyl acetate. The catalase crystals and each specimen grid were placed consecutively into the same position of the specimen holder and photographed (EM film 4489; Carestream Health Inc., Rochester NY) in the eucentric optical plane of the TEM using 60 K nominal magnification. The photographic negatives were scanned at 1200 dpi for further use.

### Selecting and Measuring Rosette CSCs in the Replicas

A total of 497 images of rosette CSCs were picked by hand from relatively flat areas of the replicas with continuous shadowing. Somewhat indistinct or apparently 5-lobed rosette CSCs [about 9% of the total in this study, similar to prior observations in a related moss^27^] were included. Measurements were made of 324 six-lobed rosette CSCs (ImageJ; http://imagej.nih.gov/ij/). Diameter was estimated two ways: (a) based on the perimeter of the smallest circle that could contain all six lobes and circular geometry; and (b) based on an irregular hexagon defined by the outer center edge of individual lobes and regular hexagon geometry, which avoided the need for precise measurement of each side. These are only useful estimates because some rosette CSCs appeared slightly elliptical, possibly due to small tilts in the membrane plane.

A total of 300 perimeter measurements (6 lobes of 50 rosette CSCs) were made to determine the average single lobe area. The CSCs analyzed included those with the smallest and largest estimated circular diameters and 48 others selected at random across the range of circular diameters (Fig. 2). Lobe perimeters were traced by hand and converted to area in Image J, followed by determining average lobe area for each CSC.

### Class Averaging of Rosette CSC Images

The rosette CSC images were black/white inverted to match the typical input of the averaging software, centered within 31.6 nm square boxes, aligned, classified, and averaged using three different programs: EMAN2 ( http://blake.bcm.edu/emanwiki/EMAN2), RELION (REgularised LIkelihood OptimisatioN, as implemented in EMAN2) and ISAC/SPARX (Iterative Stable Alignment and Clustering/Single Particle Analysis for Resolution Extension; http://sparx-em.org/sparxwiki/sxisac). ISAC/SPARX generates class averages that are reproducible in multiple classification trials. This iterative routine placed 313 rosette CSCs into six stable classes: the first 4 classes (including 193 images) were generated with the img_per_grp parameter set to 99 and the last two classes (including 120 images) were generated when img_per_grp was set to 60 and the thld_err value to 10. EMAN2 and RELION were set to generate six classes for consistency with the ISAC/SPARX results. Hand measurements were repeated on the clear class average images, and the center-to-center spacing of opposite lobes was measured. A total of 916 distinct individual lobes were picked with 10.3 nm square boxes, aligned, classified, and averaged into 6 or 12 classes using EMAN2.

### Predicting and Modeling the Structure of an 8 TMH Region from CESA

Corresponding to topological predictions for CESAs^41^ (Supplementary Fig. S1), we generated an 8 TMH computational model for the first spatial comparison to the rosette CSC images. This 8 TMH model included 323 amino acids representing the predicted TMH in cotton GhCESA1 and short linkers between them, excluding the N-terminus, central cytosolic domain, and the C-terminus of the native protein (Supplementary Fig. S4). From ten good quality models (Supplementary Table S1) and two structural predictions (Supplementary Fig. S5), a preferred model was used in molecular dynamics simulations with a lipid-bilayer membrane to assess the validity and stability of the TMH monomer. CHARMM-GUI ( http://www.charmm-gui.org) was used to generate a DOPC lipid membrane. Explicit water molecules, Na^+^, and Cl^−^ were added to build the solvated system with 63,135 atoms, followed by system minimization. After gradual heating (to 300 K), equilibration MD (20 ns) and production runs (100 ns) were carried out using Amber14^42^. Parameters included Langevin dynamics with the NPγT ensemble with semi-isotropic pressure coupling to an external bath with a relaxation time of 1.0 ps where the collision frequency = 1.0, random seed, and surface tension at 10.0 dyne/cm. The force fields used were ff99SB^43^ for the protein, lipid11^44^, and TIP3P^45^ for the water. The SHAKE algorithm^46^ constrained the covalent bonds involving hydrogen atoms, allowing for a 2.0 fs time step. The particle-mesh Ewald method was used to treat all electrostatic interactions with a real space cutoff of 10 Å. The last 100 ns-MD trajectories are depicted in Supplementary Fig, S6. Using this monomeric model, the trimer was arranged using ZDOCK^47^ followed by local minimizations to obtain a more stable structure.

### Comparing an Average Image of the Rosette CSC with Various Oligomers of the 8 TMH Model

Alternative homo-oligomers of the 8 TMH model were compared with the average FF-TEM image. We initially observed that the area occupied by dimeric models of the TMH regions created by M-ZDOCK^47^ ( http://zlab.umassmed.edu/m-zdock/) was similar to simply omitting the monomer from the trimer, and this option was used for further work to generate the most direct comparison with the trimer area. Supplementary Figure S3 establishes the usefulness of M-ZDOCK for generating other oligomers. Adobe Photoshop 6 ( http://www.adobe.com/products/photoshopfamily.html) was used to determine cross-sectional areas. The dimeric to hexameric oligomers of the 8 TMH model were replicated six times, centered by hand within Adobe Photoshop 6 on each lobe in ISAC/SPARX class average 2, and rotated to approximate the shape of the averaged lobes and minimize clash, if applicable. Each oligomer model was then blurred to 2.5 nm in Sculptor^48^ ( http://sculptor.biomachina.org/), with an isosurface threshold for rendering solid surfaces set to 5.4 to extend to the edges of the Van der Waals model of the protein. Sculptor’s image capture function was used to generate bitmap images scaled the same as the FF-TEM image.

### Analysis of Potential Energy and Size of Various Oligomers of a Seven TMH CESA Model

Only seven TMH may exist in CESA if predicted TMH5 instead exists on the cytosolic side of the membrane^28^. By manually specifying BcsA as a main template, we generated a 7 TMH model with structural similarity to BcsA in the TMH region. This was refined by MD, then docked to an optimized model of the CESA cytosolic domain^30^, which differed from the original model^7^ only by improved folding of the peripheral P-CR and CSR domains. Dimeric through hexameric oligomers of the modeled 7 TMH CESA monomer were docked with SymmDock^49^,^50^( http://bioinfo3d.cs.tau.ac.il/SymmDock/). This Monte Carlo method generated a pool of 20000 conformations for each oligomeric assembly, which were sorted based on lowest interfacial contact energy and overall TMH vertical alignment, as must occur when CESA spans the membrane. The selected conformations for each assembly were further refined using explicit water MD simulations. For system sizes less than 1 million atoms, minimization was carried out with the Amber14 suite^42^ using a combination of 1000 steps of steepest descent, and the conjugate gradient method was performed followed by multistep heating the system to 300 K at a constant volume within 40 ps. A constant pressure MD run was performed where the long-range electrostatic interactions were calculated by particle mesh Ewald summation^51^ with a 0.00001 tolerance of Ewald convergence, and the non-bonded interactions were truncated at 9 Å. The temperature was maintained at 300 K using a Berendsen thermostat^52^. The SHAKE algorithm^46^ was used to constrain hydrogen-atom vibrations. Simulations were performed using the Amber14 package with GPU accelerated PMEMD code^53^ ( http://ambermd.org/) on multiple graphics processing unit (GPU) cards (GeForce GTX 780; NVIDIA Corp., Santa Clara, CA). The all atom model consisted of the proteins and neutralizing ions and water molecules, which cumulatively represented 100000-500000 atoms per assembly. The lipid membrane was excluded in the simulations due to system size constrains. Short MD simulation runs were performed for up to 1 ns, which allowed the protein assembly to relax at room temperature while the TMH remained vertically aligned. The potential energies of the oligomers were estimated using the NAMD energy module with the Visual molecular dynamics molecular graphics program^54^ (VMD), which included both bonded and non-bonded energy terms, then normalized by the number of monomers in each oligomer.

### Six-fold Assemblies of Various Modeled CESA Oligomers

Six-fold assemblies of the relaxed oligomers were generated to mimic assembly possibilities for the rosette CSC. Dimer and trimer assemblies were docked with SymmDock as described above to generate a pool of 2000 conformations, followed by minor manual adjustments in helical alignment. Tetramer, pentamer, and hexamer assemblies were generated manually due to system size limitations in SymmDock. Explicit MD simulations on each six-fold assembly were performed using the combination of NAMD 2.10 software^55^ and Amber suite. The protein assembly, neutralizing ions and water molecules cumulatively represented 1.6 million to over 3 million atoms per system. These six-fold assemblies were parametrized for the Cornell force field from the Amber suite, minimized using conjugate gradient with NAMD, and run in parallel on four GPU cards as described above. Post minimization, heating, and equilibration steps were performed using Amber14/PMEMD as described above on a single GPU card that supports simulations of up to 5 million atoms (GeForce GTX Titan X; NVIDIA Corp., Santa Clara, CA). The potential energies per monomer within each of the six-fold assemblies were determined as described above for the individual oligomers.

### Demonstration of Triangularity in a CESA Cystosolic Domain Trimer and Comparison of its SAXS-derived Model to the Rosette CSC

A solution (3.5 μl of a 5 μg/ml solution) of recombinant AtCESA1 catalytic domain^18^ was adsorbed on carbon-coated glow-discharged grids, which were washed and stained simultaneously with 10 drops of 1% (wt/vol) of freshly prepared uranyl formate. Air-dried samples were analyzed in a TEM (Tecnai T12; FEI, Hillsboro, OR) operating at 120 keV using a dose of ~20 e^−^/Å^2^ and a defocus value of 0.5–2.5 μm. Images were acquired at 68 k magnification (1.45 Å/pixel on the specimen) with a 4 k × 4 k charge-coupled device (CCD) camera (Eagle; FEI, Hillsboro, OR). Images were evaluated and 138 particles were picked within 32.5 nm (or 224 pixels) square boxes using EMAN2, which then generated six class averages (Supplementary Fig. S7)

Given SAXS data consistent with a trimer^18^, GASBOR^56^ was used to generate a set of *ab initio* models imposing P3 symmetry followed by averaging and filtering using DAMAVER and DAMFILT^25^ with filtering threshold set to 9 for the final model^57^. This filtered average model is considered to be the ‘most probable’ model ( http://www.embl-hamburg.de/biosaxs/manuals/damaver.html). The shape of the SAXS cytosolic model was compared to the cytosolic portion of the 7 TMH CESA model by structural superimposition with SUPCOMB ( http://www.embl-hamburg.de/biosaxs/supcomb.html)^31^. For spatial comparison of the model to the FF-TEM or negative stain average images, atomic measurements were made in PyMOL ( http://www.pymol.org) and converted to pixel values with the GNU image manipulation program ( http://www.gimp.org/). Representations of protein models were rendered in Blender ( http://www.blender.org).

## Additional Information

**How to cite this article**: Nixon, B. T. *et al*. Comparative Structural and Computational Analysis Supports Eighteen Cellulose Synthases in the Plant Cellulose Synthesis Complex. *Sci. Rep.* **6**, 28696; doi: 10.1038/srep28696 (2016).

## Supplementary Material

Supplementary Information

## Figures and Tables

**Table 1:** Dimensions of replicated rosette CSCs and individual lobes measured on original images and class averages.

Type of image analyzed	Geometry	Perimeter (nm)	Diameter (nm)	Lobe Area (nm^2^)
Original images (n = 324)	Circle	78.4 ± 4.9	24.9 ± 1.6	39.9 ± 6.5 (n = 300)
	Hexagon	74.8 ± 4.5	21.4 ± 1.3	
Class averages of entire rosette CSCs				
ISAC/SPARX (n = 6)	Circle	76.8 ± 2.5	24.3 ± 0.8	37.6 ± 3.3 (n = 36)
	Hexagon	78.0 ± 3.1	22.5 ± 0.9	
RELION (n = 2)	Circle	78.2 ± 0.4	24.9 ± 0.1	42.0 ± 2.7 (n = 12)
	Hexagon	79.9 ± 1.5	23.1 ± 0.4	
EMAN2 (n = 6)	Circle	76.4 ± 2.3	24.3 ± 0.7	36.7 ± 4.9 (n = 36)
	Hexagon	78.7 ± 1.9	22.7 ± 0.5	
Class averages of individual lobes				
6 classes	–	–	–	40.6 ± 4.4 (n = 6)
12 classes	–	–	–	38.7 ± 2.9 (n = 12)
Means				
Means for Original Images		76.6	23.1	39.9
Means for Class Averages		78.0	23.6	39.1
Overall Means		77.3	23.4	39.5

Diameter was estimated from the perimeter using two geometries. ‘Lobe Area’ is the average value for one lobe. ± values indicate standard deviation; n values for perimeter and diameter are shown in column 1 and indicate entire rosette CSCs; n values for lobe area are shown in column 5 and indicate individual lobes or lobe image averages measured.

**Table 2:** Predicted cross-sectional area of a 7 or 8 TMH region in various predicted oligomers.

*In silico* model	TMH cross-sectional area for various oligomers (nm^**2**^)				
	dimer	trimer	tetramer	pentamer	hexamer
8 TMH only	28.7	41.9	47.2	65.2	82.1
7 TMH CESA (including the large central domain)	30.4	44.4	49.4	62.1	88.0

The TMH cross sectional areas in various oligomers of two alternative computational models are shown. ‘8 TMH’ indicates a model including only eight alpha helical regions and short linkers between them. In contrast, ‘7 TMH CESA’ indicates a model inclusive of seven TMH and the large central cytosolic domain. These dimensions can be compared to the range of single lobe areas derived from original images or image averages: 37.6–42.0 nm^2^ (overall mean of 39.5 nm^2^; see Table 1).

**Table 3:** Predicted potential energies per monomer of various oligomers of the 7 TMH CESA model.

Assembly level	Potential energy per monomer (kcal/mol × 10^−5^)				
	dimer	trimer	tetramer	pentamer	hexamer
Single oligomer	−1.03	−1.65	−1.51	−1.59	−1.69
Six-fold assembly of oligomers	−1.42	−2.50	−1.89	−1.68	−2.20
