# Supplementary material for: Comparative Structural and Computational Analysis Supports Eighteen Cellulose Synthases in the Plant Cellulose Synthesis Complex
Source: Sci Rep. 2016 Jun 27;6:28696. doi: 10.1038/srep28696 (PMC4921827; doi:10.1038/srep28696)
Supplement: Supplementary Information [file srep28696-s1.pdf]

SUPPLEMENTARY INFORMATION

Comparative Structural and Computational Analysis Supports Eighteen Cellulose Synthases in the Plant Cellulose Synthesis Complex

B. Tracy Nixon[a,1,2], Katayoun Mansouri[b,1], Abhishek Singh[c,1], Juan Du[a,1], Jonathan K. Davis[b, 1], Jung-Goo Lee[c], Erin Slabaugh[b], Venu Gopal Vandavasi[d], Hugh O'Neill[d,], Eric M. Roberts[e], Alison W. Roberts[f], Yaroslava G. Yingling[c,2], Candace H. Haigler[b,2]

[a]Department of Biochemistry and Molecular Biology, Pennsylvania State University, PA 16802, USA
[b]Department of Crop Science and Department of Plant and Microbial Biology, North Carolina State University, Raleigh, NC 27695, USA
[c]Department of Materials Science and Engineering, North Carolina State University, Raleigh, NC 27695, USA
[d]Oak Ridge National Laboratory, Oak Ridge, TN 37831, USA
[e]Department of Biology, Rhode Island College, Providence, RI 02908, USA
[f]Department of Biological Sciences, University of Rhode Island, Kingston, RI 02881, USA

Notes on Authors:
[1]The first five authors contributed equally to this research.
Present Address for K.M.: Duke University, Cell Biology, Box 3011, Durham, NC 27710

[2]Address correspondence to candace_haigler@ncsu.edu (FF-TEM and general aspects), yara_yingling@ncsu.edu (computational modeling), btn1@psu.edu (single particle EM, SAXS).

SUPPLEMENTARY METHODS

Generating the Cartoon of the Rosette CSC in the Context of the FF-TEM Process

3D representations in Figure 1 were based on the trimeric assembly of the 7 TMH CESA model. The cartoon was created by symmetric assembly of six trimers in Blender (http://www.blender.org), using the average FF-TEM image (ISAC SPARX class average 2; Fig. 3) to position the TMH region of the trimers. Lipid bilayers were generated by randomly distributing a single 1-palmitoyl 2-oleoyl phosphatidyl choline molecule from a MD-simulated gel-phase lipid bilayer (Heller et al., 1993). Meshconv (http://www.cs.princeton.edu/~min/meshconv/) and Meshlab (http://www.3d-coform.eu/index.php/tools/meshlab) were used for conversion of 3D mesh files generated by PyMOL (https://www.pymol.org/) to formats compatible with Blender.

Aquaporin-4 as a Control for Methods Used to Analyze Rosette CSC Structure and Lobe Oligomerization.

Rotary shadowing could potentially add to the size of a protein oligomer measured in a replica prepared by freeze fracture electron microscopy (FF-TEM) according to the formula: (measured film thickness) x cotangent $(\theta) \times 2/\pi$, where θ is the shadowing angle and π is the rotation factor (Ruben, 1989). To test whether this effect is likely to have occurred in the replicas used in this study, we compared the size of the tetrameric protein aquaporin-4 (AQP4) as derived from high quality FF-TEM replicas (similar to the ones we prepared; Rash et al., 2004) with the actual size of the assembled transmembrane helices (TMHs) as derived from its crystal structure (Hiroaki et al., 2006). Each monomer of AQP4 contains eight membrane-embedded helical segments, with six of them passing through the membrane as TMHs. Stable tetramers as well as larger orthogonal arrays form in membranes, so that AQP4 is easily recognized in FF-TEM replicas, as

confirmed by immunolabeling (Papadopoulos and Verkman, 2013). The shape of tetrameric AQP4 as derived from FF-TEM images (Supplementary Fig. S1A, originally published as Fig. 9E; Rash et al., 2004) was compared with its atomic protein structure (Supplementary Fig. S1B) as derived from two dimensional (2D) crystals (Hiroaki et al., 2006). The shape in Supplementary Figure S1A originated from four-fold (0°, 90°, 180°, 270°) rotation and reinforcement of FF-TEM images of AQP4 arrays (Rash et al., 2004). This required a choice of the 0° position, and it was done prior to the availability of data on the tilt of the tetramer within the larger AQP4 arrays (Hiroaki et al., 2006). The slight offset of the 0° position compared to reality resulted in bright and dim areas (at the free corners of the AQP4 tetramer) within the reinforced shape shown in Supplementary Figure S1A. Therefore, the perimeter of the reinforced shape, inclusive of areas of high and low reinforcement, was traced in Supplementary Figure S1C to aid visibility.

To overlay the two structural representations, Figure 9E of Rash et al., 2004 was converted to a bitmap image in which the 4.5 nm arrow spanned 219 pixels (48.67 pixels/nm). The AQP4 tetramer structural representation was created in PyMol (https://www.pymol.org/) using crystallographic symmetry data from PBD ID 2D57 and rotated to match the orientation relative to neighboring tetramers in the 2D crystal lattice (Hiroaki et al., 2006). This is expected to be highly similar to the *in vivo* lattices observed in FF-TEM (Rash et al., 2004). To match the scales of the two structural representations, a 7.45 nm atom-to-atom distance was calculated in PyMol between the C4 of phenylalanine 117 from diagonally opposite monomers. In a bitmap representation of the crystal structure, this distance corresponded to 596 pixels (80 pixels/nm). The crystal structure representation was then scaled by a factor of 0.581 to match the FF-TEM image.

The appropriately sized atomic model was overlaid on the rotationally reinforced FF-TEM shape, showing a good fit between them (Supplementary Fig. S1D). This comparison showed that an ultra-thin metal shadow in a FF-TEM replica did not appreciably increase the apparent dimensions of AQP4, an example of an oligomeric transmembrane protein. Therefore, class averages of particles imaged by FF-TEM can be expected to approximate the cross-sectional area of the assembled TMHs of multiple CESAs within one lobe of the rosette CSC without concern about potential enlargement due to the shadowing metal.

To show that M-ZDOCK generates reasonable oligomers, residues Gln32 to Pro254 of the monomer of rat AQP4 in PDB ID 2D57 were used to generate a tetramer, which was then superimposed on the biological tetramer built from the symmetry intrinsic to the space group (90, $P42_12$) of the rat AQP4 crystal structure. The biological tetramer was made using two functions in SwissPDBViewer (http://spdbv.vital-it.ch/): Tools/Build Crystallographic Symmetry and Tools/Translate Layer Along Unit Cell. The structural alignment was then made using the Fit/Iterative Magic Fit function, selecting the 'all atoms' criterion to define the atoms to include in the alignment.

Modeling of CESA Transmembrane Helices with and without BcsA as a Template

The GhCesA1 sequence was truncated to 323 amino acids to include the eight predicted TMHs (Supplementary Fig. S4) and submitted for three-dimensional structure prediction using RaptorX (Kalberg et al., 2012). The RaptorX protein structure prediction server accommodates even low sequence homology between the experimental sequence and available templates through selection and use of multiple distantly related template proteins and a nonlinear scoring function with a probabilistic-consistency algorithm. We ran RaptorX two times, with and without the structure of BcsA from *R. sphaeroides*, PDB 4HG6, included as a template, to generate ten good quality models (Supplementary Table S1) and two structural predictions (Supplementary Fig. S5). In Table S1, some templates (e.g. NADH-quinone oxioreductases with the same PDB identifier) appear more than once because slightly different regions of CESA were matched with the same template (see different p-values and probability scores) during the two runs of RaptorX. The predictions shown in the left and right panels of Supplementary Figure S5 were based on models 1,2,5,6,7 or 3,4,8,9,10, respectively of Supplementary Table S1.

Supplementary Table S1. Ten representative models of predicted TMH regions of CESA generated with the RaptorX server.

Model number	P-value	Score	uGDT/ GDT	uSeqID/ SeqID	PDB Template	Description of template	Snapshots of CESA TMH models
1	4.8e-05	157	88/34	22/8	3rkoC	NADH-quinone oxidoreductase	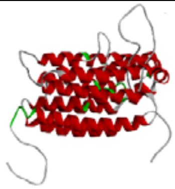
2	6.3e-05	153	77/30	24/9	3rkoB	NADH-quinone oxidoreductase	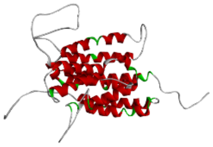
3	6.9e-05	142	64/22	21/7	3rkoC	NADH-quinone oxidoreductase	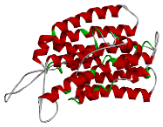
4	9.9e-05	137	55/19	21/7	3rkoB	NADH-quinone oxidoreductase	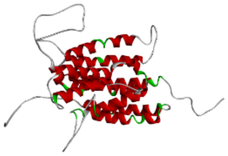
5	1.2e-04	144	103/37	21/7	4av3A	sodium-pumping pyrophosphatase	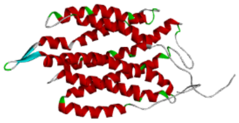
6	1.5e-04	141	125/46	33/12	3eh3A	Cytochrome c oxidase	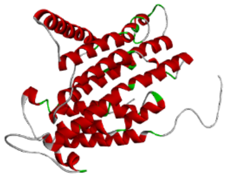
7	1.9e-04	137	101/39	20/8	2yevA	Cytochrome c oxidase	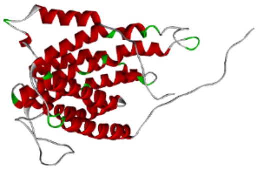
8	2.4e-04	126	85/33	31/12	4p00A	Cellulose Synthase	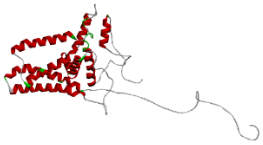
9	1.8e-04	130	64/24	25/9	4he8I	NADH-quinone oxidoreductase	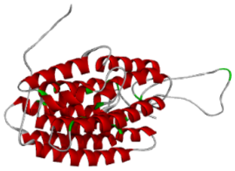

10	1.2e-04	135	59/21	25/9	4he8F	NADH-quinone oxidoreductase	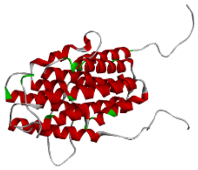

RaptorX server (http://raptorx.uchicago.edu/; Källberg et al., 2012)

Smaller P-values indicate higher quality models, and P ≤ E-03 is a good indicator for mainly alpha helical proteins.

Score is the alignment score falling between 0 and the (domain) sequence length, with 0 indicating the worst.

GDT is Global Distance Test and uGDT is the unnormalized GDT score, which measures the absolute model quality. For a protein with >100 residues, uGDT>50 is a good indicator.

uSeqID is the number of identical residues in the alignment. SeqID is uSeqID normalized by the protein (or domain) sequence length and multiplied by 100. Higher uSeqID (SeqID) is better.

Supplementary Table S2. Dimeric to hexameric assemblies of the 7 TMH CESA model. The scale varies between oligomers and views.

Oligomer	Top View	Side View	Bottom View
Dimer	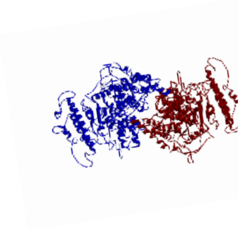	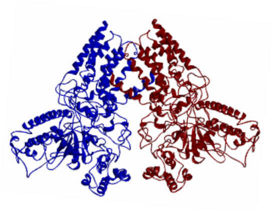	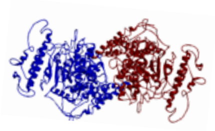
Trimer	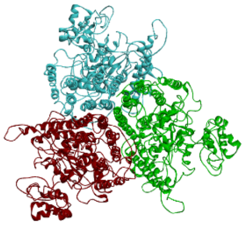	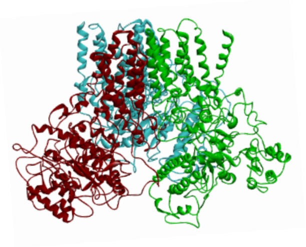	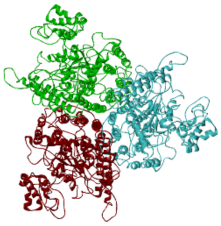
Tetramer	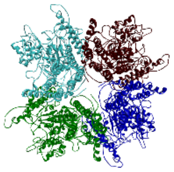	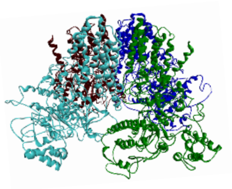	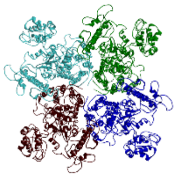
Pentamer	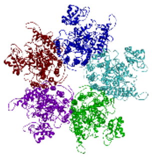	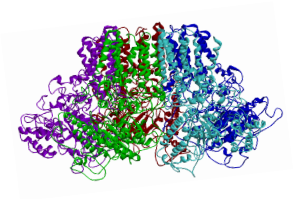	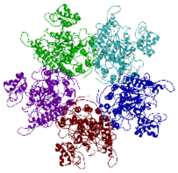
Hexamer	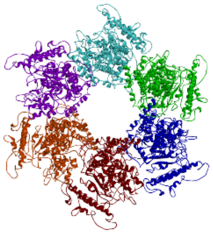	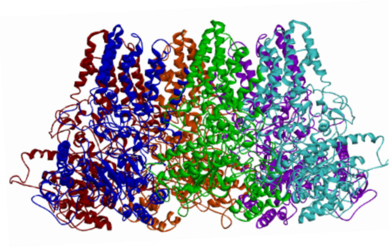	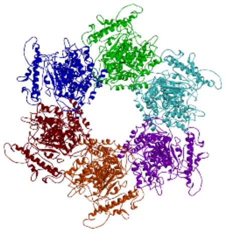

```
                                                       TMH1
GhCESA1 EQQMEDKPAPDASQPLSTIIPIPKSRLAPYRTVIIMRLIILGLFFHYRVTNPVDSAFGLW 202
AtCESA1 SNGEELQMADDTRLPMSRVVPIPSSRLTPYRVVIILRLIILCFFLQYRTTHPVKNAYPLW 304
PpCESA8 PNGPDLPIMDESRQPLSRKIPIASSRINPYRMVIVIRLVVLAFFLRYRILHPVEGACGLW 306
McCESA1 NGGNDLPLMDESRQPLSRKVDFNMGLIQPYRLMIVIRLVVLAFFLRYRILNPAPSR-PLW 353
MdCESA1 E-SADLPSMDESRQPLSRKIPYPSALINPYRLVIIIRFFVVGLFLSWRLTTPVPDAWWLW 291
SmCESA1 GNGPDLPIMDEARQPLSRKVPIPSSRINPYRMIIVIRLVILGFFFRYRIMNPVRDAYPLW 314
             :     ::  *:*  :    . : *** :*::*:.:: :*: :*   *.     **
          TMH2
GhCESA1 LTSVICEIWFAFSWVLDQFPKWYPVNRETYIDRLSARYEREGEPDELAAVDFFVSTVDPL 262
AtCESA1 LTSVICEIWFAFSWLLDQFPKWYPINRETYLDRLAIRYDRDGEPSQLVPVDVFVSTVDPL 364
PpCESA8 ITSVVCEIWFAVSWILDQFPKWLPIQRETYLDRLSLRYEKPGEPSQLVNVDVYVSTVDPL 366
McCESA1 MTSVICEIWFAVSWILDQFPKWMPINRETYLDRLNLRFEKEGEPSQLQAVDLFVSTVDPE 413
MdCESA1 LFSVICEVWFAFSWILDQFPKWMPLRRETYMDRLSLRFERKNEPSGLAPVDLFISTVDPA 351
SmCESA1 LTSIICEIWFALSWILDQFPKWLPIERETYLDRLSLRYEKDGEPSQLASVDVYVSTVDPM 374
        : *::**:***.**:******* *:.****:***  *:::  **. *  **.::*****
```

```
                                       TMH3
GhCESA1 LSRHCPLWYGFGGGRLKWLQRLAYINTIVYPFTSLPLIAYCSLPAICLLTGKFIIPTLSN 782
AtCESA1 LSRHCPIWYGYH-GRLRLLERIAYINTIVYPITSIPLIAYCILPAFCLITDRFIIPEISN 889
PpCESA8 LSRHCPLWYGYG--RLKCLERLAYINTTIYPLTSLPLVAYCTLPAVCLLTGKFIIPTISN 901
McCESA1 LSRHCPIWYGWSGSRLKLLQRLAYINTVVYPFTAFPLLAYCTLPAICLLTNQFIIPEISS 938
MdCESA1 LSRHCPIWYGWKANKLKVLQRMAYINTVVYPFTSFPLIIYCILPAVCLFTNSFIIPQLDT 882
SmCESA1 LSRHCPLWYGYG-GRLKWLERFAYINTTVYPLTSIPLVAYCTLPAVCLLTGKFIIPEISN 903
        ******:***:   :*: *:*:***** :**:*::**: ** ***.**:*  **** :..
          TMH4                                 TMH5
GhCESA1 LASVLFLGLFLSIIVTAVLELRWSGVSIEDLWRNEQFWVIGGVSAHLFAVFQGFLKMLAG 842
AtCESA1 YASIWFILLFISIAVTGILELRWSGVSIEDWWRNEQFWVIGGTSAHLFAVFQGLLKVLAG 949
PpCESA8 LDSLWFISLFMSIFITGILEMRWSGVGIDEWWRNEQFWVIGGVSAHLFALFQGLLKVFAG 961
McCESA1 LNSLWFIALFISIFACAFLEMRWSGVGMEEWWRNEQFWVIGGVSSHLYAVFQGLLKVLAG 998
MdCESA1 VALFYFVALFICIFATGVLEMRWSKVSMTEWWRNEQFWVIGGTSAHLFAVFQGLLKVLAG 942
SmCESA1 FASLWFISMFVSIFATAILEMRWSNVGIEEWWRNEQFWVIGGVSSHLFAVFQGLLKVLAG 963
          . *: :*:.*   ..**:*** *.: : ***********.*:**:*:***:**::**
                                       TMH6
GhCESA1 IDTNFTVTAKAA-DDADFGELYIVKWTTLLIPPTTLLIVNMVGVVAGFSDALNKGYEAWG 901
AtCESA1 IDTNFTVTSKATDEDGDFAELYIFKWTALLIPPTTVLLVNLIGIVAGVSYAVNSGYQSWG 1009
PpCESA8 IDTNFTVTSKTG-EDEDFGELYTLKWTSLLIPPTTLLLFNMVGVVAGISDAINNGYSAWG 1020
McCESA1 IDTNFTVTAKAADDGEAYADLYLFKWTSLLIPPTTLIIINLIGAVAGVANAINNGYDQWG 1058
MdCESA1 IDTNFTVTAKQVDEGE-FAELYVFKWTSLLIPPLFLLILNGLGIASGVAQMVNTGSGAWG 1001
SmCESA1 IDTNFTVTSKAT-DDEEFGELYTLKWTTLLVPPTTLLIINLVGVVAGLADAINSGYQSWG 1022
        ********:*   :   :.:** .***:**:**  :::.* :* .:*.:  :*.*   **
           TMH7                      TMH8
GhCESA1 PLFGKVFFSFWVILHLYPFLKGLMGRQNRTPTIVVLWSVLLASVFSLVWVRINPFVSTAD 961
AtCESA1 PLFGKLFFALWVIAHLYPFLKGLLGRQNRTPTIVIVWSVLLASIFSLLWVRINPFVDANP 1069
PpCESA8 PLFGKLFFAFWVIVHLYPFLKGLMGRQNRTPTIVIVWSILLASIFSLLWVRIDPFLPKST 1080
McCESA1 PLFGKLFFAFWVVVHLYPFLKGLMGKSNRTPTLIIVWSVLLASIFSLLWVKINPFTNTTN 1118
MdCESA1 QLFGKLFFSFWVIVHLYPFMKGLGGRSQKIPTLVIVWSVLLSSIFSLLWVRIDPFTAAPS 1061
SmCESA1 PLFGKLFFAFWVIVHLYPFLKGLMGRQNRTPTIVIVWSILLASIFSLLWVRIDPFLPKTQ 1082
          ****:**::**: *****:*** *:.:: **::::**:**:*:***:**:*:**

GhCESA1 STTVSQSCISIDC-  974
AtCESA1 NANNFNGK--GGVF 1081
PpCESA8 GPNLVRCG--LTC- 1091
McCESA1 GPALVQCGIRC--- 1129
MdCESA1 GPTLQQCGVSC--- 1072
SmCESA1 GPHLQQCG--LNC- 1093
```

Supplementary Figure S1. Protein sequence alignment of the predicted TMH regions of diverse CESAs. The large catalytic domain sequence was deleted at the position of the horizontal black line. MEMPACK (SVM Prediction of TM Topology and Helix Packing, http://bioinf.cs.ucl.ac.uk/psipred; Jones, 1999), which uses Hidden Markov Models, predicted the same eight TMHs for six CESAs from algae and plants with a wide phylogenetic distribution. Octopus (http://octopus.cbr.su.se/; Viklund and Elofsson, 2008) confirmed the result for GhCESA1 (GenBank: AAB37766.1). There was a high sequence conservation between isoforms in the membrane-spanning region. The six CESAs were from: *Gossypium hirsutum* (cotton GhCESA1, AAB37766.1, associated with secondary wall synthesis); *Arabidopsis thaliana* (AtCESA1, AAC39334.1, associated with primary wall synthesis); *P. patens* (moss PpCESA8, ABI78961.1); *Selaginella moellendorffii* (a seedless vascular plant, spikemoss, SmCESA1, EFJ17343.1); and two Zygnematalean algae, *Mesotaenium caldariorum* (McCESA1, AAM83096.1) and *Micrasterias denticulata* (MdCESA1, ADE44904.1).

Supplementary Figure S2 (two pages)

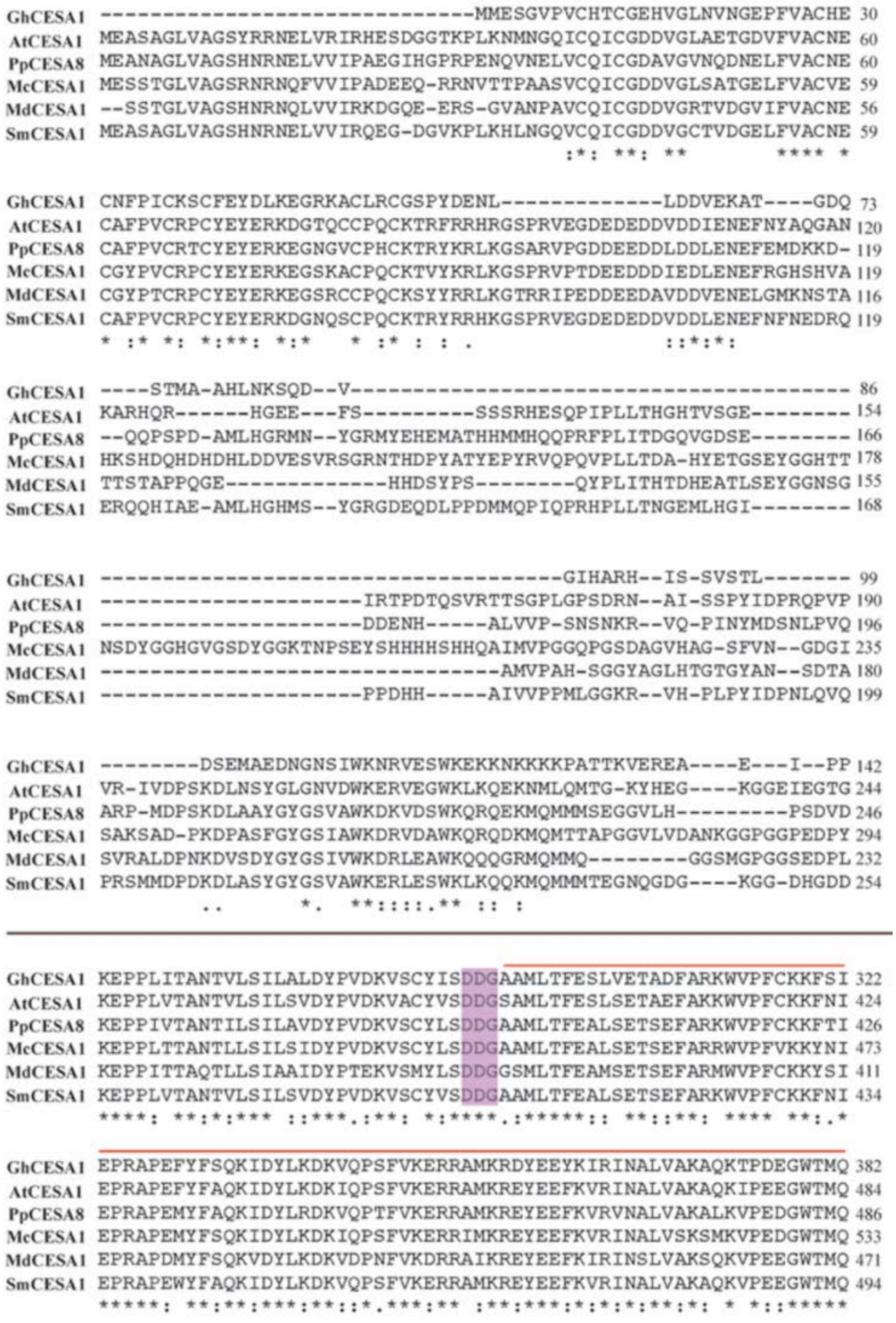

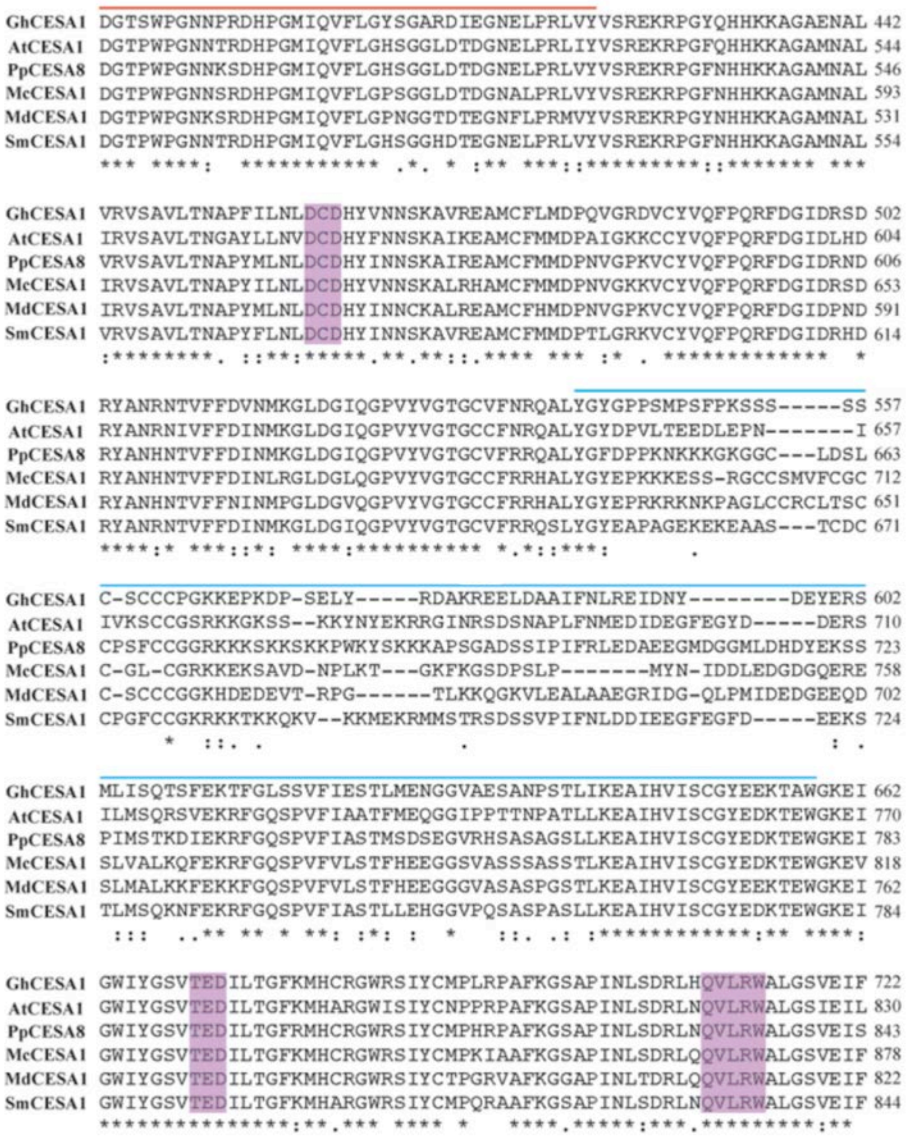

Supplementary Figure S2. Protein sequence alignment of the majority of the N-terminal domain (up-stream of TMH1) and the majority of the large catalytic domain (between TMH2 and TMH3) of diverse CESAs. The alignment shows the variability in the N-terminal region of different CESA isoforms, whereas the catalytic region is highly conserved except in the CSR region. The region containing TMH1 and TMH2 was deleted at the point of the horizontal black line. Four conserved motifs related to catalysis are highlighted with purple blocks. The P-CR and CSR regions are highlighted with red and blue lines, respectively. The organisms and protein identifiers are listed in Supplementary Figure S1.

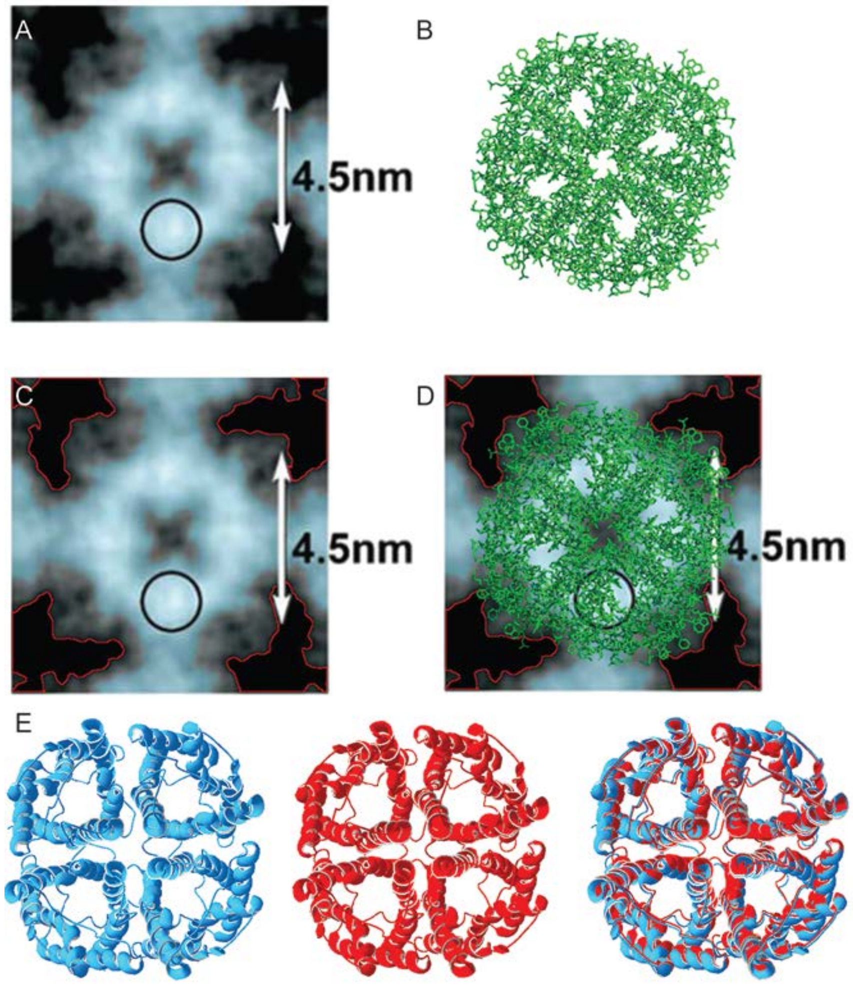

Supplementary Figure S3. Aquaporin-4 as a control for methods used to analyze rosette CSC structure and oligomerization. See Supplementary Methods for further explanation. In **(A-D)** we show that the methods we used do not add detectable thickness of shadowing metal to the size of transmembrane protein oligomers as observed in FF-TEM. This was accomplished by overlaying a crystallographic tetramer of aquaporin-4 (AQP4) with a rotationally-reinforced shape derived from replicas prepared by freeze-fracture transmission electron microscopy. (**A)** The tetrameric shape derived from rotational reinforcement of FF-TEM images of rat AQP4 arrays. The circle in the original image indicated the estimated diameter of a single alpha helix (reproduced from Figure 9E of Rash et al. 2004). (**B)** Atomic model of a tetramer of rat AQP4 (PDB ID 2D57), tilted as in the two dimensional crystalline lattice (Hiroaki et al. 2006) and scaled to match the 4.5 nm arrow in (A). **(C)** Red trace around the periphery of the FF-TEM-derived tetramer in (A); (**D)** Overlay of the atomic model on the FF-TEM-derived image shows a good fit between them. See the Supplementary Methods for additional details. **(E)** M-ZDock, as used to generate CESA oligomers in Figure 3E, was able to correctly generate the tetramer of AQP4. The biological tetramer is shown on the left (blue), one of several similar tetramers generated from the monomer by M-ZDock is shown in the middle (red), and the overlay of 6608 backbone and side chain atoms for residues Gln32-Pro254 is shown on the right (RMSD 1.75 Å).

Supplementary Figure S4 (see the legend at the bottom of the page)

A truncated assembly of 323 amino acids of GhCESA1 inclusive of the eight predicted transmembrane helices

```
QPLSTIIPIPKSRLAPYRTVIIMRLIILGLFFHYRVTNPVDSAFGLWLTSVICEIWFAFS
WVLDQFPKWYPVNRHCPLWYGFGGGRLKWLQRLAYINTIVYPFTSLPLIAYCSLPAICLL
TGKFIIPTLSNLASVLFLGLFLSIIVTAVLELRWSGVSIEDLWRNEQFWVIGGVSAHLFA
VFQGFLKMLAGIDTNFTVTAKAADDADFGELYIVKWTTLLIPPTTLLIVNMVGVVAGFSD
ALNKGYEAWGPLFGKVFFSFWVILHLYPFLKGLMGRQNRTPTIVVLWSVLLASVFSLVWV
RINPFVSTADSTTVSQSCISIDC
```

Secondary structure of the GhCESA1 transmembrane helical region predicted by the Octopus Server

```
oooooooooooooooMMMMMMMMMMMMMMMMMMMMMiiiiiiMMMMMMMMMMMMMMMMMM
MMMooooooooooooooooooooooooooooooooooooMMMMMMMMMMMMMMMMMMMMM
iiiiiiiiiiiMMMMMMMMMMMMMMMMMMMMMoooooooooooooooooooMMMMMMMMM
MMMMMMMMMMMMiiiiiiiiiiiiiiiiiiiiiiiiiMMMMMMMMMMMMMMMMMMMMMoo
oooooooooMMMMMMMMMMMMMMMMMMMMMiiiiiiiiiiMMMMMMMMMMMMMMMMMMMM
Moooooooooooooooooooooo
```

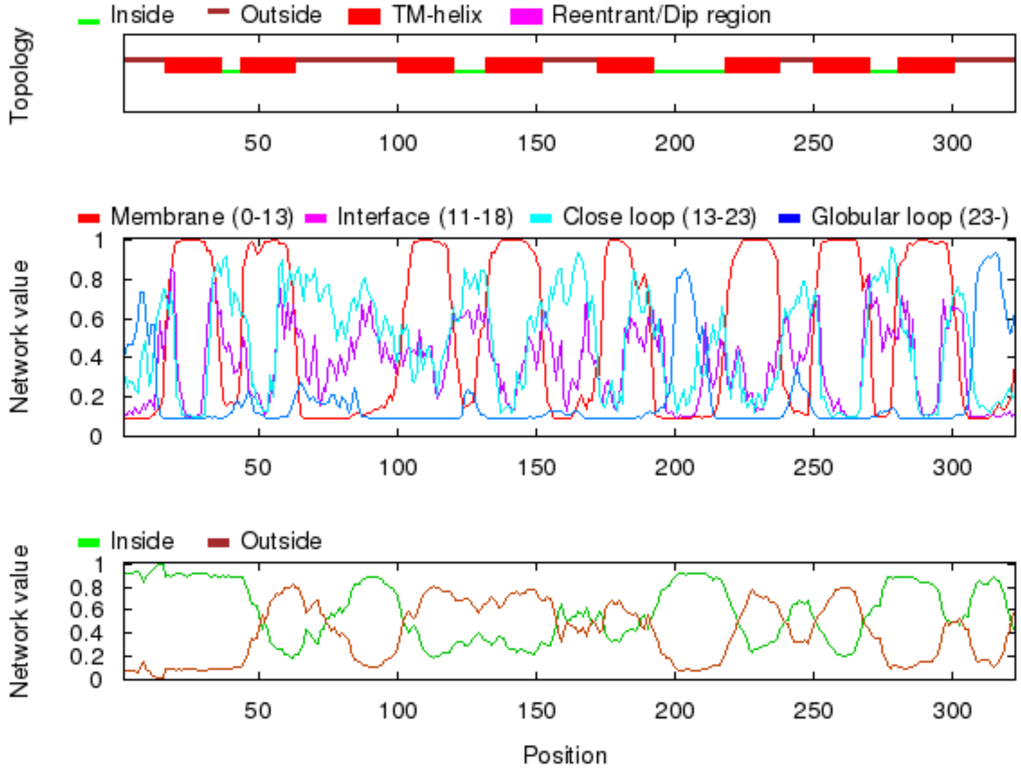

Supplementary Figure S4. Secondary structure predictions about the putative 8 TMH region of a CESA. The assembled sequence of the predicted TMH region from GhCESA1 is shown, along with secondary structure predictions from the OCTOPUS server (http://octopus.cbr.su.se/). In the text output, ‘o’, ‘i’. or ‘M’ represent peptide regions that were predicted to be outside, inside, or within the membrane, respectively, as also shown diagrammatically. The realistic protein orientation relative to the membrane is often not represented by computational predictions of TMH regions.

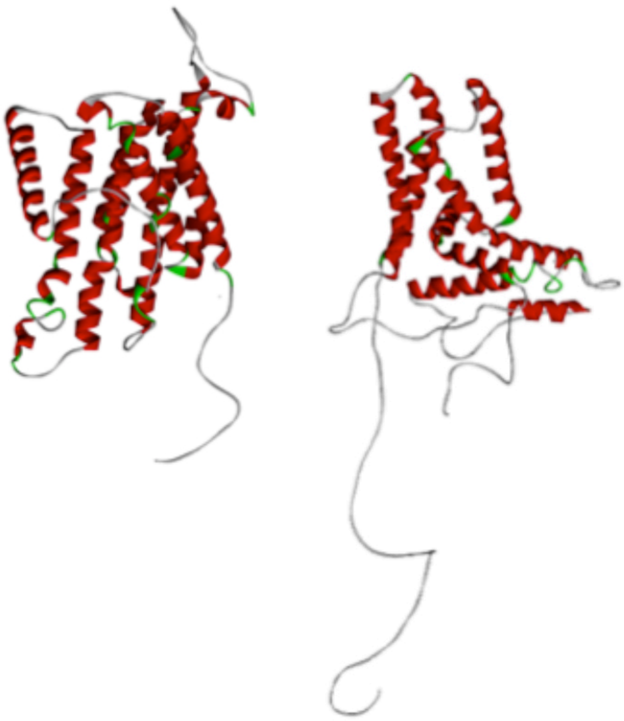

Supplementary Figure S5. Two structural models of the truncated putative 8 TMH region of a CESA, as derived from the RaptorX server. See Supplementary Methods for further explanation. The left panel shows the predicted structure of a putative 8 TMH region of GhCESA1 without the use of BcsA from *R. sphaeroides*, PDB 4HG6, as a template (Morgan et al., 2013). The right panel shows the result with BcsA as a template. The prediction based on BcsA consisted of only 4 TMHs due to the limited homology between the TMHs of BcsA and plant CESAs (Slabaugh et al., 2014). The predicted 8 TMH model on the left was used for further work.

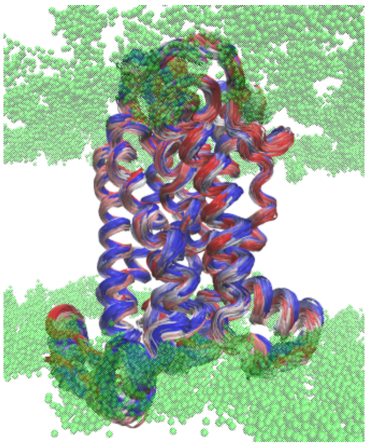

Supplementary Figure S6. Molecular Dynamics trajectories of the 8 TMH model. The N-terminus, C-terminus, and the large central catalytic domain were not included in this model, as explained further in the main text. For the side-view of the TMH region of GhCESA1, the beginning, middle, and end of the trajectory are shown in red, white, and blue, respectively. The P atoms of the lipid, DOPC, are shown in green.

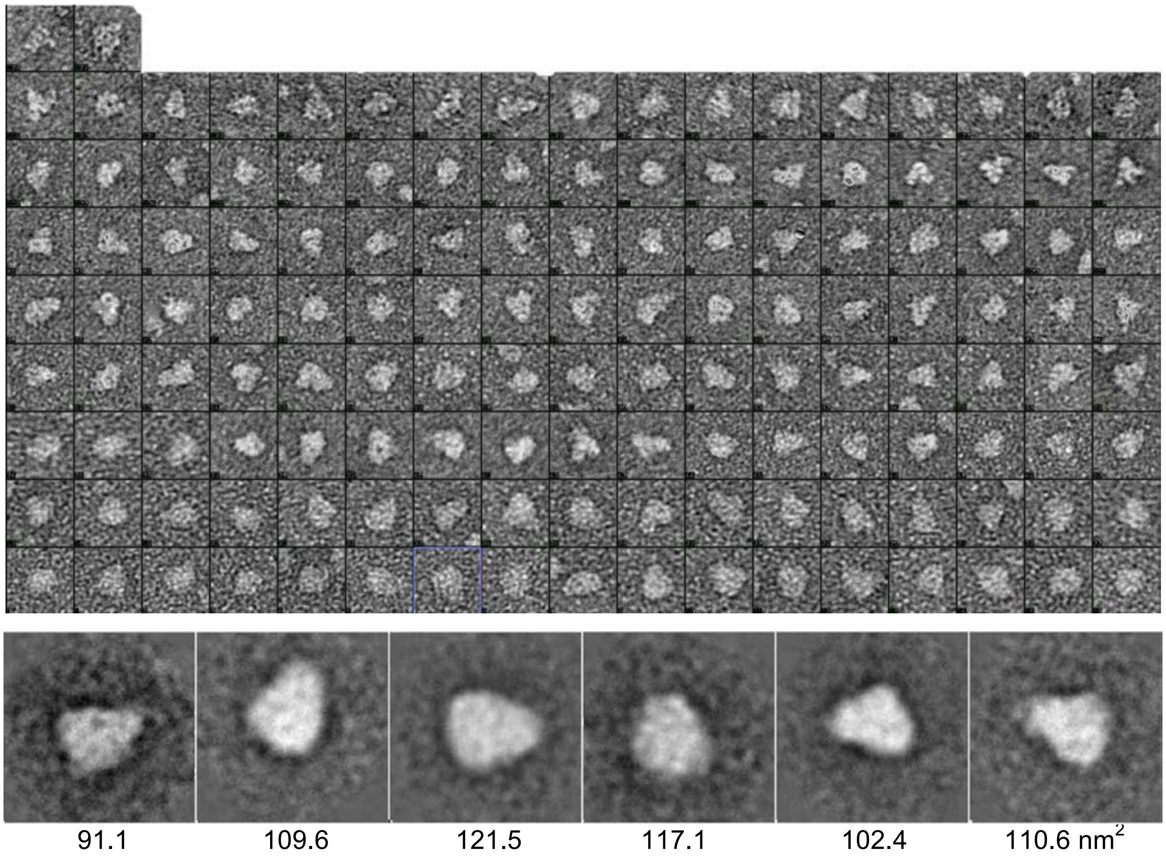

Supplementary Figure S7. Negative stained images of the heterologously expressed and purified cytosolic domain of AtCESA1.
(Top) A gallery of 138 negatively stained, randomly oriented particles is shown. The protein solution was generated as described in Vandavasi et al., 2015.
(Bottom) Six class averages generated from the gallery showing different particle orientations on the TEM grids. The cross-sectional areas (nm^2) of each particle are shown below the images: the average value was 108.8 ± 10.8 nm^2. The second class average was chosen for comparison to models in the main text because its cross-sectional area was nearest the mean.
The scale for both panels is indicated by the 32.5 x 32.5 nm boxes.

SUPPLEMENTARY REFERENCES

Heller, H., Schaefer, M., and Schulten, K. (1993). Molecular dynamics simulation of a bilayer of 200 lipids in the gel and in the liquid-crystal phases. J. Phys. Chem. 97: 8343–8360.

Hiroaki, Y, Tani, K., Kamegawa, A., Gyobu, N., Nishikawa, K., Suzuki, H., Walz, T., Sasaki, S., Mitsuoka, K., Kimura, K., Mizoguchi, A., and Fujiyoshi, Y. (2006). Implications of the aquaporin-4 structure on array formation and cell adhesion. J. Mol. Biol. 355: 628-639.

Jones, D.T. (1999). Protein secondary structure prediction based on position-specific scoring matrices. J. Mol. Biol. 292: 195–202.

Källberg, M., Wang, H., Wang, S., Peng, J., Wang, Z., Lu, H., and Xu, J. (2012). Template-based protein structure modeling using the RaptorX web server. Nature Protocols 7: 1511–1522.

Morgan, J.L.W., Strumillo, J., and Zimmer, J. (2013). Crystallographic snapshot of cellulose synthesis and membrane translocation. Nature 493: 181–186.

Papadopoulos, M.C., and Verkman, A.S. (2013). Aquaporin water channels in the nervous system. Nature Rev. 14: 265-277.

Rash, J.E., Davidson, K.G.V., Yasumura, T., and Furman, C.S. (2004). Freeze fracture and immunogold analysis of Aquaporin-4 (AQP4) square arrays, with models of AQP4 lattice assembly. Neuroscience 129: 915-934.

Ruben, G.C. (1989). Ultrathin (1 nm) vertically shadowed platinum-carbon replicas for imaging individual molecules in freeze-etched biological DNA and material science metal and plastic specimens. J. Electron Micr. Tech. 13:335-354.

Slabaugh, E., Davis, J.K., Haigler, C.H., Yingling, Y.G., and Zimmer, J. (2014). Cellulose synthases: new insights from crystallography and modeling. Trends Plant Sci. 19: 99–106.

Vandavasi, V.G., Putnam, D.K., Zhang, Q., Petridis, L., Heller, W.T., Nixon, B.T., Haigler, C.H., Kalluri, U., Coates, L, Langan, P., Smith, J.C., Meiler, J., and O'Neill, H. (on line, Nov 10, 2015). A structural study of recombinant CESA1 catalytic domain of Arabidopsis thaliana cellulose synthesis complex: evidence for CESA trimers. Plant Physiol. pp.01356.2015.

Viklund, H., and Elofsson, A. (2008). Improving topology prediction by two-track ANN-based preference scores and an extended topological grammar. Bioinformatics 24: 1662–1668.
